# Synthesis and Characterization of Type II Ge-Si Clathrate Films for Optoelectronic Applications

**DOI:** 10.3390/ma17020504

**Published:** 2024-01-20

**Authors:** Rahul Kumar, Shiori Kurita, Fumitaka Ohashi, Tamio Iida, Hitoe Habuchi, Tetsuji Kume

**Affiliations:** 1 Department of Electrical and Computer Engineering, National Institute of Technology (KOSEN), Gifu College, Gifu 501-0495, Japan; iida@gifu-nct.ac.jp (T.I.); habuchi@gifu-nct.ac.jp (H.H.); 2 Faculty of Engineering, Gifu University, Gifu 501-1193, Japankume.tetsuji.c7@f.gifu-u.ac.jp (T.K.)

**Keywords:** clathrate, thin film synthesis, type II SiGe clathrate

## Abstract

Type II inorganic clathrates consist of cage-like structures with open frameworks, and they are considered promising materials due to their unique properties. However, the difficulty of synthesizing phase-pure and continuous films has hindered their application in practical devices. In this report, we demonstrate the synthesis of type II SiGe clathrate films through the thermal decomposition of a Na-deposited amorphous SiGe film on a sapphire substrate in a high vacuum. The as-prepared films of type II SiGe clathrates showed uniform growth and were evaluated for their structural and optical properties. Morphological studies conducted using a scanning electron microscope showed the presence of cracks on the film surface.

## 1. Introduction

The increasing demands for enhanced performance and energy efficiency in electronic devices have brought the limitations of the traditional diamond structured Si/Ge into the spotlight. Numerous structural forms of Si/Ge have been actively investigated, such as the amorphous state, nanostructures, superlattices, etc. Si/Ge is a part of the group IV elements which are among the most investigated materials with various structures, primarily due to the flexibility of the covalently bonded sp^3^ hybridized framework; additionally, it can be stabilized in the clathrate structure [1,2,3,4,5,6,7]. The clathrate structures of group IV elements are commonly termed as inorganic clathrates, and the most common structures of inorganic clathrates include types I and II. The clathrate structure mainly consists of a cage-like open framework that can trap guest species (commonly alkali or alkaline earth metals) inside, leading to the chemical formulas of M_8_E_46_ and M_24_E_136_ (M: guest species; E: Si, Ge, or Sn) for type I and type II structures, respectively, for fully occupied clathrates [1,8,9,10,11]. The type II clathrate structure is formed by the face-sharing of 16 dodecahedral (E_20_) cages and 8 hexakaidecahedral (E_28_) cages, and it crystalizes in a face-centered cubic lattice (space group: *Fd*$\bar{3}$*m*). The guest species act as the templates for the synthesis of the clathrate structure and impart unique features due to their guest–host interactions. The guest species trapped inside the polyhedra of this expanded volume structure donates charge carriers to the host framework, leading to metal-like behavior with a high guest occupancy of binary and defect-free (e.g., Zintl defects) clathrates [12,13,14]. Furthermore, upon a reduced guest occupancy (<8 for type II clathrate unit cell) or alloying with suitable elements, a semiconducting nature is observed with exciting optoelectronic properties. Superconductivity was reported for Ba*_x_*Na*_y_*Si_46_ clathrates [15,16,17,18]. Hence, clathrates have attracted significant attention due to their wide range of applications, such as in metals, semiconductors, superconductors [15,16,18], and phonon glass electron crystals [19], among others.

A type II clathrate structure allows for the removal of guest species without affecting its stability, leading to unique features such as a semiconductor with a direct band gap. Type II Si and Ge clathrates with low guest occupancies exhibit direct band gaps of 1.9 eV [20] and 0.7 eV [21], respectively, in contrast to the indirect band gap of diamond-structured Si and Ge. Hence, Si- and Ge-based type II clathrates are investigated for applications in photovoltaics [9,22,23], thermoelectric devices [21], and Li-ion batteries [24,25,26,27]. Recently, alloy clathrates, in which the host framework is made up of more than one group IV element (Si, Ge, or Sn), have been actively investigated due to their potential applications as thermoelectric materials, optoelectronic materials, etc. Among them, alloyed clathrates of Ge and Si are of great importance [28,29]. Bandgap tuning by alloying Si and Ge for type II clathrates has been theoretically calculated to be in the range of 0.81 eV to 1.74 eV [21] and has been experimentally reported to be in the range of 0.6 eV [30] to 1.9 eV [9]. Hence, band gap tuning in the visible spectrum will be ideal for applications in LEDs and thin-film solar cells. The properties of the clathrates can also be modified flexibly by alloying with other elements such as group III elements, group V elements, etc. [31].

The type II clathrate has been synthesized in various form factors, such as powders and thin films [32,33], by employing a range of synthesis methods. For powder samples, type II clathrates have been synthesized mainly using a two-step thermal decomposition method [34,35,36], an ionic liquid method [30], and an electrochemical method [26,37,38,39]. On the other hand, for the film form, a two-step thermal decomposition method, primarily derived from the powder synthesis method, was used predominantly [3,20,23,32,33]. Recently, our team has reported a single-step thermal decomposition method using a specially designed setup [31,40,41]. To control the guest concentration in the type II clathrate films, various techniques have been employed. Among them, lowering the guest concentration through prolonged annealing under a high vacuum has been widely investigated. Other techniques include electric field application coupled with annealing in the inert atmosphere for the removal of Na in a type II Ge clathrate film [32]. Recently, Vollondat et al. demonstrated the removal of a Na guest from a type II Si clathrate film by annealing in an iodine vapor, whereas a fully occupied type II Si clathrate film was achieved through an extended exposition to sodium vapor [20]. For optical property analysis or to realize its potential in practical devices, the film form is preferred over powder. However, the main challenges in film fabrication involve obtaining uniformly grown film samples with good surface features. The presence of cracks on the surface or non-uniform growth hinders the accurate measurement of the film properties.

The type II SiGe clathrate has been investigated through theoretical studies [21,28]. Martinez et al. and Baranowski et al. have investigated the type II Si/Ge clathrates in powder form [22,42]; however, there are currently no reports on experimental studies of type II SiGe clathrate films to the best of our knowledge. In this study, the fabrication of a type II SiGe film (5%, 10%, and 15% of Si molar composition) on a sapphire substrate with a film feature suitable for investigating the optical and electrical properties was attempted. A single-step thermal decomposition method was employed [40,41], which requires less time for film fabrication and results in improved surface features compared to the two-step method [32]. The as-prepared film was characterized by X-ray diffraction and Raman scattering spectroscopy, confirming the synthesis of a type II SiGe film. Scanning electron microscopy of the film surface revealed small cracks, and an optical transmission study was used to estimate the absorption coefficient spectra.

## 2. Materials and Methods

Amorphous SiGe (a-SiGe) film, which was co-sputtered using RF sputtering from Si and Ge targets and deposited on a sapphire substrate (20 × 10 mm^2^), served as the starting material. The sapphire substrate was selected for this study because it is optically transparent, electrically insulating, and stable at a higher temperature. The sputtering chamber was evacuated down to 10^−5^ Pa, and the process pressure was maintained at 5 Pa with an Ar flow rate of 50 SCCM during sputtering. The substrate temperature was maintained at 400 °C. The power settings of the Ge and Si targets were adjusted to obtain various molar ratios of Si in the a-SiGe film. The Si/Ge ratio was investigated through an EDX measurement of the sputtered film, and a-SiGe films with molar ratios of 5% Si, 10% Si, and 15% Si were confirmed.

The starting material (a-SiGe film) was transferred inside a specially designed chamber, namely the Portable Vacuum Evaporation and Annealing System (pVEAS). The details of the pVEAS setup can be obtained from previous reports created by our research group, which facilitate the deposition of Na on the precursor film in a high vacuum and subsequent annealing in the same chamber [40,41]. The a-SiGe film was placed on the sample holder facing downwards in the chamber, and Na lumps (Nippon Soda, Tokyo, Japan, 99.95%) of small sizes were placed in the tungsten basket located directly below the sample. The handling of Na was performed inside a glove box filled with dry argon under safety precautions. The chamber was sealed, and then transferred outside and connected to the vacuum system and IR lamp heater. The chamber was evacuated using the fitted rotary and turbo molecular pumps below ~10^−4^ Pa of the dynamic vacuum. Subsequently, Na was evaporated from the tungsten basket by applying an external electric field to the tungsten basket through the connected feedthroughs while maintaining the chamber at a high vacuum. The Na-deposited a-SiGe film was then annealed by the IR lamp with a power output corresponding to 250 °C while still maintaining the high vacuum to obtain a type II SiGe clathrate film. Upon the completion of annealing, the sample was allowed to cool to room temperature naturally.

For the synthesis of pure type II Ge clathrate film using pVEAS, amorphous Ge (a-Ge) film prepared by RF sputtering served as the precursor. In pursuit of good-quality films of type II Ge clathrate on a sapphire substrate, we fine-tuned the synthesis parameters. The process involved depositing Na onto the a-Ge film at a substrate temperature of 230 °C, followed by a subsequent annealing phase at the same temperature for 3 h.

The as-prepared film was investigated through X-ray diffraction (XRD) by Rigaku Smartlab (Cu *K_α_* radiation, *λ* = 0.154 nm) in grazing incidence (GIXRD) mode at an angle of incidence of *ω* = 2°. Rietveld refinement of the XRD data was carried out to estimate the lattice parameters, atomic positions, and Na occupancy in the polyhedral cages, using PDXL 2 software package (version 2.1.3.4) by Rigaku. Raman scattering spectroscopy was performed using JASCO (Tokyo, Japan), NRS-2100 G (laser source: 532 nm). The film surface morphology, film thickness, and elemental composition were investigated by top-view analysis, cross-sectional analysis, and energy-dispersive X-ray spectroscopy (EDX), respectively, using a field emission scanning electron microscope (Hitachi Hi-Tech, Tokyo, Japan, S-4800). An accelerating voltage of 5.0 kV was used. Optical properties of the clathrate films were analyzed using a single-beam Fourier transform infrared spectrometer (Perkin Elmer, Shelton, CT, USA, Spectrum 100) and a dual-beam ultraviolet-visible near-infrared (UV-vis-NIR) spectrometer (JASCO, Tokyo, Japan, V-670). All measurements were carried out at room temperature.

## 3. Results

**Figure 1:**
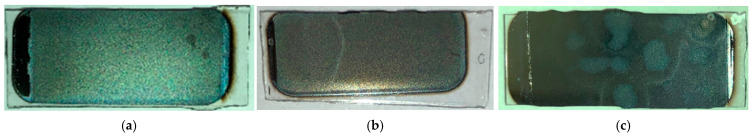
Photograph of as-prepared type II SiGe clathrate film on sapphire substrate (20 × 10 mm^2^): (**a**) 5% Si (sample 1), (**b**) 10% Si (sample 2), and (**c**) 15% Si (sample 3).

The photographs in Figure 1 depict the as-prepared film with varying Si/Ge concentrations, which was prepared at an annealing temperature of 250 °C for 8 h. A visual inspection suggested the uniform and homogenous growth of the film without any visible surface defects. The film samples appeared translucent when placed in front of a white light source, in contrast to the semi-transparent nature of the type II Ge clathrate film prepared at the same annealing temperature. The optimization of the synthesis parameters for a type II Ge clathrate film, such as Na deposition at a higher substrate temperature of a-Ge film and annealing at a lower temperature (230 °C for 3 h), resulted in the suppression of the semi-transparent nature, and an almost opaque film was achieved.

### 3.1. Structural and Morphological Characterization

**Figure 2:**
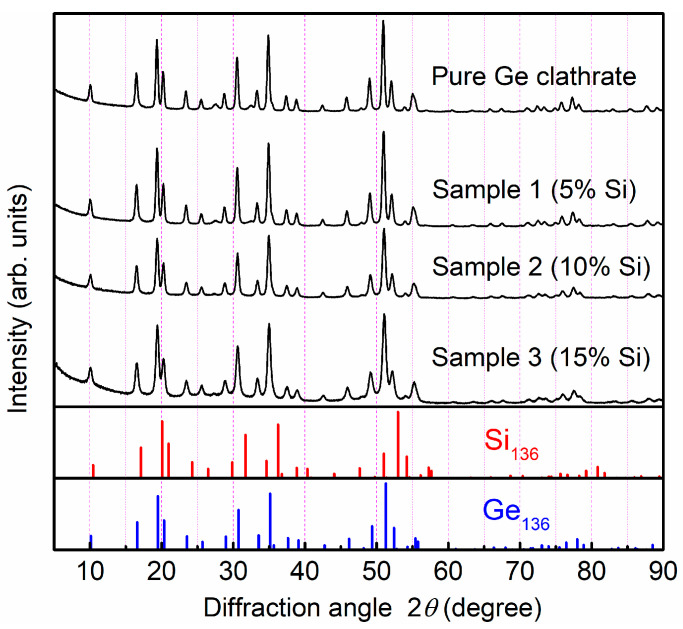
GIXRD pattern of the type II Ge clathrate and type II SiGe clathrate films obtained with incident angle of *ω* = 2°.

The X-ray diffractograms obtained from the as-prepared film samples with varying Si ratios are shown in Figure 2. Observed reflections were indexed to the Na*_x_*Ge_136_ and Na*_x_*Si_136_ phases. The as-prepared films were polycrystalline in nature, and no epitaxial growth was observed. No impurity phase, such as diamond-structured Ge or Si, was detected, suggesting the successful synthesis of type II SiGe clathrate films. When comparing the XRD results with that of the pure type II Ge clathrate, peak broadening with an increase in the Si ratio was observed. Broad background peaks were clearly identifiable at 2*θ*~20° and 52°, suggesting the presence of an amorphous Si-like phase for samples with higher Si ratios. The global profiles of the peak intensities suggested that there were low Na concentrations in all of the as-prepared film samples, which are typically identifiable by the small relative intensity of the peak at ~10°. The exact estimation of the Na contents was carried out through a Rietveld analysis of the XRD data, which is discussed in a later section.

**Figure 3:**
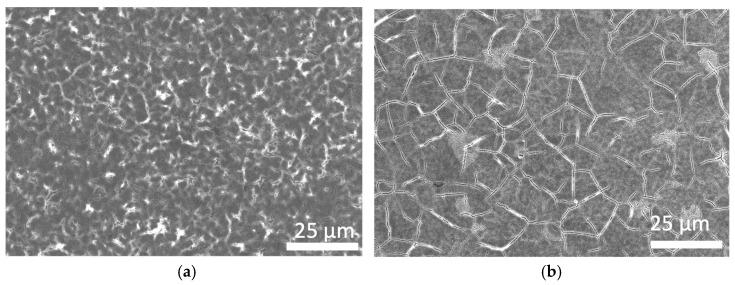
Plan-view FESEM micrograph of type II SiGe clathrate film obtained from (**a**) sample 1 (5% Si) and (**b**) sample 2 (15% Si).

**Figure 4:**
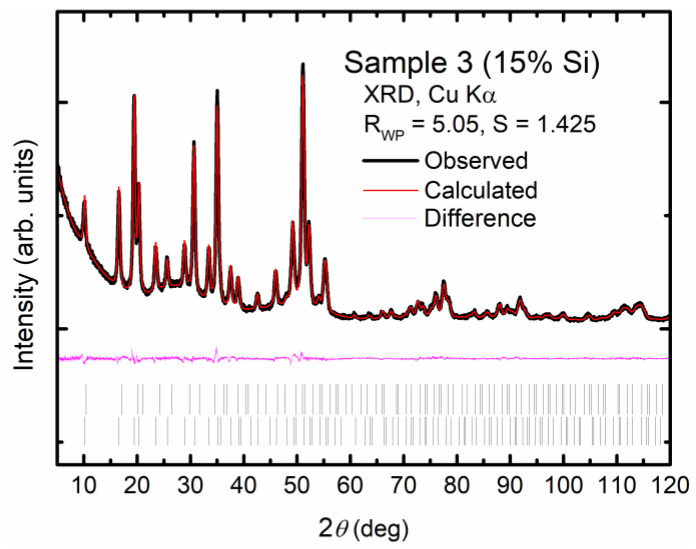
Rietveld refinement result of XRD data obtained from type II SiGe clathrate film, sample 3 (15% Si). The black line corresponds to the measured pattern, the red line corresponds to the calculated pattern, the pink line is the difference, and the bars (|) depict the Bragg’s diffraction peaks.

The top surface FESEM micrographs of the as-prepared films are presented in Figure 3. Small cracks on the surfaces of the type II SiGe clathrate films were observed, suggesting a granular structure. The total area of the surface cracks appeared to be reduced in the samples with increases in the Si ratio. The EDX results of the as-prepared film samples, shown in Table 1, confirm the Si/Ge molar ratios of 5% Si in sample 1, 10% Si in sample 2, and 15% Si in sample 3, which were found to be the same as those of the starting material (a-SiGe film). The Rietveld refinement of the XRD data was performed by fixing the Si/Ge molar ratio obtained from the EDX measurement, and the refinement fitting curves of sample 3 are shown in Figure 4. The lattice constants (*a*) of the as-prepared samples were estimated as 15.2070(3) Å, 15.1929(3) Å, and 15.1828(5) Å for sample 1, sample 2, and sample 3, respectively. The guest occupancies at the 8*b* and 16*c* sites were used to calculate the Na contents. The amounts of Na (*x*) in the as-prepared type II SiGe clathrate (Na*_x_*(Si*_y_*Ge_1-*y*_)_136_) films were estimated as 1.00(28), 1.02(24), and 0.93(32) for sample 1, sample 2, and sample 3, respectively. The Rietveld refinement results of sample 3 are summarized in Table 2.

**Figure 5:**
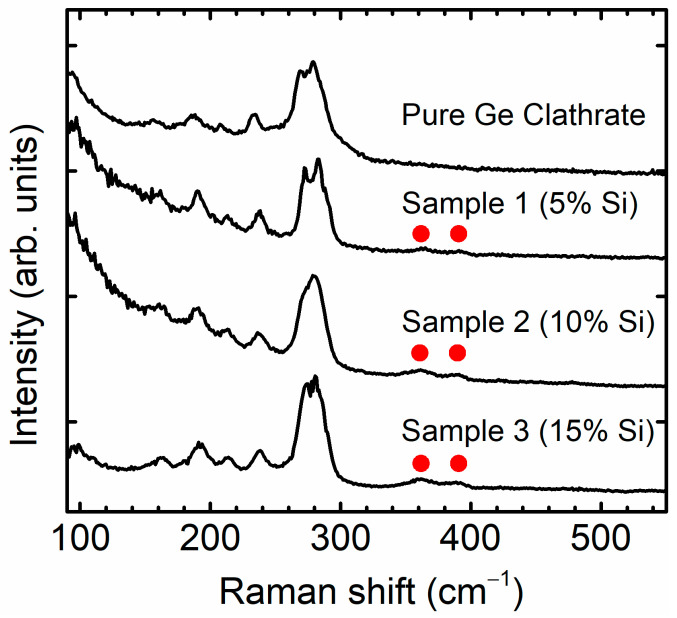
Raman scattering spectra obtained from type II SiGe clathrate film synthesized on the sapphire substrate.

The Raman scattering spectra obtained from the as-prepared film samples are shown in Figure 5. The major peaks obtained were assigned to the vibrational modes of the Na*_x_*Ge_136_ phase. No characteristic peaks in the diamond phase of Si/Ge were detected. A systematic blue shift of the major peaks was observed for the samples with increasing Si ratios. This blue shift phenomenon is often correlated with a decrease in crystallinity or material subjected to compressive stress [20]. Hence, the presence of an amorphous phase, supported by the XRD measurement results, and lattice strain, due to substitutional effects of the Ge lattice with smaller Si atoms, were attributed to the observed blue shift of the Raman scattering spectra. Additionally, weak intensity peaks centered at 360 cm^−1^ and 390 cm^−1^ were observed, as indicated by the red dots; these are discussed in the subsequent section.

### 3.2. Optical Characterization

**Figure 6:**
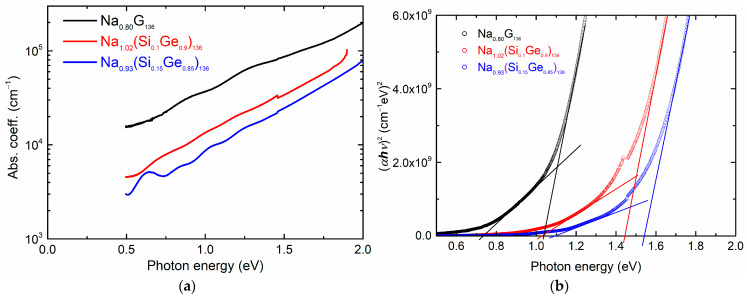
Optical properties investigated using transmission studies: (**a**) absorption coefficient spectra of type II SiGe clathrate films synthesized on the sapphire substrate; (**b**) band gap estimation via Tauc plot under direct band gap scheme.

The optical properties of type II SiGe clathrate films were investigated through transmission measurements. Figure 6a shows the absorption coefficient spectra of the as-prepared film samples that were compared with those of the type II Ge clathrate to investigate the effects of Si inclusion. The absorption coefficient was calculated using a formula, −ln⁡(T)/d, where *T* represents the transmittance and *d* represents the film thickness. The thicknesses of the film samples were estimated using cross-sectional FESEM measurements, and they were 334 nm, 1454 nm, and 1302 nm for the Na_0.80_Ge_136_, Na_1.02_(Si_0.1_Ge_0.9_)_136_, and Na_0.93_(Si_0.15_Ge_0.85_)_136_ film samples, respectively. Compared to the type II Ge clathrate, a blue shift in the spectra was observed for the type II SiGe clathrates, which increased with the Si concentration. The estimation of the optical band gap using a Tauc plot under the direct band gap scheme is shown in Figure 6b. An interpretation of the Tauc plot appeared complicated due to the lack of a clear absorption edge. However, a careful observation revealed that two absorption edges can be drawn from the spectra, which are depicted by the solid lines in Figure 6b.

## 4. Discussion

The as-prepared type II clathrate films appeared opaque with no visible defects, such as pin holes, suggesting uniform film growth. The XRD peaks were indexed to the type II SiGe clathrate phase, and no impurity phase was detected. Upon a comparison with the XRD peak profile of the pure type II Ge clathrate, peak broadening was observed, which was attributed to the a-Si/Ge-like states present at the top surface or at the periphery of the grain boundaries. The presence of disordered Si states in the type II Si clathrate was extensively investigated in previous reports [12,43]. The FESEM micrographs revealed the presence of small cracks on the film surface of the as-prepared film sample in the micrometer range, which can be attributed to the fast rate of phase transformation (from the intermediate zintl phase to the type II clathrate), inherent surface defects on an RF-sputtered a-SiGe film, etc. An EDX analysis for the Si:Ge ratio estimation revealed that the ratios for sample 1, sample 2, and sample 3 were 5%, 10%, and 15% of Si, respectively, which were consistent with the values of the starting material (a-SiGe). However, the Na contents *x* (in Na*_x_*(Si*_y_*Ge_1-*y*_)_136_) calculated using an EDX analysis appeared to be overestimated, as the type II SiGe clathrate samples with high Na contents resulted in *x* > 24. This observed phenomenon was speculated to originate due to the inhomogeneous distribution of Na at the surface and the bulk of the film sample, which needs to be verified through further investigations. For the Rietveld refinement of the XRD data, the Si/Ge molar ratio obtained from the EDX measurement was fixed. The refinement fitting parameters appeared unaffected by small changes in the Si molar ratio of the type II SiGe clathrate. The lattice constant (*a*) decreased slightly with the increase in the Si ratio, which is in good agreement with previously reported results [31,40]. The estimation of the Na contents (*x*) resulted in the empirical formulas of Na_1.00_(Si_0.05_Ge_0.95_)_136_, Na_1.02_(Si_0.10_Ge_0.90_)_136_, and Na_0.93_(Si_0.15_Ge_0.85_)_136_ for sample 1, sample 2, and sample 3, respectively. In the Raman scattering spectra shown in Figure 5, peaks corresponding to the vibrational modes of the Na*_x_*Ge_136_ phase were observed [40,41]. Additionally, weak intensity peaks observed at ~360 cm^−1^ and 390 cm^−1^ were assigned to the Si-Ge bond vibration, which is in agreement with the reported results for the diamond-structured Si-Ge bond vibrations [44] and the theoretical calculations for the vibrational density of states of the Ge rich type II SiGe alloy clathrates [21]. It is worth mentioning that this represents the first observation of a Si-Ge bond vibration for a type II SiGe clathrate, to the best of our knowledge, and corroborates the XRD results.

The absorption coefficient spectra of the as-prepared film (Figure 6a) shifted to higher energies with an increase in the Si ratio, indicating a systematic blue shift of the spectra with the increased Si ratio in the type II SiGe clathrate. This implies that the host framework of the type II Ge clathrate was substituted by Si atoms, consequently modifying the band structure and shifting the absorption spectra. The band gap energy was estimated using a Tauc plot (Figure 6b) under the direct band gap scheme. The Tauc plot suggested two possible absorption edges, which are indicated by solid lines. When considering the theoretical calculations of the band structure for the type II SiGe clathrate, the valence band maximum and the conduction band minimum at the *L* point were indicated as a direct band gap [21,28]. Furthermore, a conduction minimum present at the Γ point was attributed to a nearly direct band gap. For the type II Ge clathrate (0% Si), the absorption edge in the lower energy range was observed at 0.74 eV, which was attributed to the direct interband transitions at the *L* point, and the absorption edge observed at 1.04 eV was attributed to the direct interband transitions at the Γ point. A similar observation of two absorption edges was reported for the type II Ge clathrate and Al-doped type II Ge clathrate films [31,40]. For the type II SiGe clathrate films, the absorption edge in the lower energy region was estimated at 1.03 eV for sample 2 (10% Si) and 1.10 eV for sample 3 (15% Si), and in the higher energy region, it was 1.45 eV for sample 2 and 1.54 eV for sample 3. A previous study on type II SiGe clathrate powder demonstrated that the absorption onset for a 15% Si sample was observed at 1.2 eV, whereas for type II Ge and Si clathrates, they were observed at 0.65 eV and 2.2 eV, respectively [42]. It is noteworthy that past reports of the band gap estimation of a type II SiGe clathrate were mainly obtained using the powder sample. In this work, we report the band gap estimation from a film sample and employ transmission measurements. Furthermore, it is speculated that the band gap tunes to the energy range of 1.3–1.4 eV, which is considered promising for photovoltaic applications, and can be achieved with the composition ratio of ~25% Si in a type II SiGe clathrate. A further investigation on properties such as the temperature-dependent electrical properties is deemed necessary for the accurate estimation of the band gap of a type II SiGe clathrate, which is ongoing in our research group and will be published in the future reports.

## 5. Conclusions

Type II SiGe clathrate films with molar ratios of 5%, 10%, and 15% of Si were synthesized on a sapphire substrate. The as-synthesized film showed uniform surface features in the visual inspection; however, the presence of cracks was revealed in a top-view FESEM investigation. The GIXRD peaks confirmed the synthesis of the type II SiGe film with small traces of an a-Si/Ge phase. The Raman scattering spectroscopy showed a blue shift in the spectra with an increase in the Si fraction, and the weak intensity peaks observed at ~360 cm^−1^ and 390 cm^−1^ were attributed to the Si-Ge bond vibration. A band gap estimation performed using the Tauc plot revealed two absorption edges, which were estimated at 1.03 eV and 1.10 eV for sample 2 and sample 3, respectively, in the lower energy range.

## Figures and Tables

**Table 1:** EDX measurement results obtained from the as-prepared type II SiGe clathrate film synthesized on the sapphire substrate.

Elements	Atomic Compositions (at. %)		
	Sample 1	Sample 2	Sample 3
C	9.68	10.48	15.43
O	20.89	2023	26.32
Na	3.36	4.68	3.62
Al	3.44	5.54	3.28
Si	2.97	5.71	7.66
Ge	59.67	53.37	43.7
Si/(Si+Ge)	0.047	0.097	0.149

**Table 2:** Structural parameters obtained through Rietveld refinement analysis. The space group was assumed as Fd-3m.

*a* (Å)	*R_wp_*	*R_exp_*	*S*	
15.1828(5)	5.05	3.54	1.425	
**Atom (site)**	***x***	***y***	***z***	**Occ**
Ge/Si (8a)	0.875	0.875	0.875	0.85/0.15
Ge/Si (32e)	0.78278(7)	0.78278(7)	0.78278(7)	0.85/0.15
Ge/Si (96g)	0.81687(4)	0.81687(4)	0.81687(4)	0.85/0.15
Na (8b)	0.375	0.375	0.375	0.020(16)
Na (16c)	0	0	0	0.048(12)

## Data Availability

All data are contained within the article.
